# Femtosecond Optical Kerr Gates in Cancerous Breast Tissue for a New Optical Biopsy Method

**DOI:** 10.21203/rs.3.rs-2829849/v1

**Published:** 2023-05-10

**Authors:** Henry Meyer, Sandra Mamani, Zhi Li, Lingyan Shi, Robert Alfano

**Affiliations:** The City College of the City University of New York; The City College of the City University of New York; University of California San Diego; University of California San Diego; The City College of the City University of New York

## Abstract

The Optical Kerr Effect was demonstrated for the first time as a new optical biopsy method to detect normal and grades of cancer of human breast tissues. The technique works by temporally tracking the various electronic and molecular processes that give rise to the nonlinear index of refraction (n_2_). The rate at which these processes populate and dissipate varies depending on the internal properties of the sample. It is shown here that in tissues, the variances in the ultrafast plasma Kerr responses that relates to the dielectric relaxation can be used as a biomarker for cancer. The relaxation of this response changes significantly between healthy and different grades of triple negative breast cancer tissues. This change can be attributed to a doubling or tripling of the tissue’s conductivity depending on the cancer grade.

## INTRODUCTION

One of most important nonlinear optical effects is the Optical Kerr effect arising from change in the index of refraction of a material to an optical pulse. A potential new optical tool in the field of optical biopsy has been found in the temporal behavior of the Optical Kerr Effect (OKE) [1–4]. Historically, the OKE has been used as a gate for traditional neat and mixed liquids, and solid materials [5,6], to measure the temporal processes associated with the Kerr Effect. Recently the OKG has been used for the first time to measure the Kerr Effect of biological tissues [7]. Human brain and animal breast tissue were shown to have a unique double peaked temporal Kerr structure in time response to a femtosecond pump laser. This structure had detectable temporal changes between the animal and human brain samples. This suggested a potential use of the technique in optical biopsy, which is studied here for the first time for cancer. The Kerr effect displays distinct cancer biomarkers in human breast tissue that arises from changes in tissue dielectric relaxation times.

A major challenge for today’s cancer patients is early detection [8]. The current optical biopsy diagnostic techniques for cancer detection are based on optical spectroscopy and microscopy [9–18]. These techniques are based on linear and nonlinear optical processes such as absorption, fluorescence and Raman scattering [9–11], which lack the necessary genomic data and biomarkers for an early diagnosis.

In this paper, human breast tissues of healthy and different grades of cancer are investigated for the first time by using the femtosecond optical Kerr Gate. This new optical biopsy diagnostic technique introduced here, can potentially overcome the current diagnostic challenges by looking for the conductivity changes as biomarker in the temporal Kerr response that arise from the relaxation associated with excessive electric charges in the structure of the tissue.

Several grades of cancerous breast tissues were studied, both non-triple-negative (non- TNBC) and triple-negative (TNBC) focusing on different grades. Breast cancer tissues were considered for this research because is the second leading cause of cancer-related death among women of color. According to global cancer data from 2012, around 1.7 million women were diagnosed with breast cancer and 521,900 women died from the disease [19]. Breast cancer is an extremely heterogeneous disease. The clinical treatment and prognosis vary considerably between patients, in particular women of color. TNBC is an aggressive breast cancer with clinically negative expression of estrogen and progesterone (ER/PR), and human epidermal growth factor receptor2 (HER2). Epidemiological studies reveal that TNBC is most prevalent in premenopausal young women under the age of 40, who account for 15–20% of all breast cancer patients [20].

Contrary to other subtypes of breast cancer, TNBC patients had a shorter survival period, with a 40% mortality rate within the first 5 years after diagnosis [21]. TNBC is highly invasive and has a high frequency distant metastasis to brain, liver and lung [22]. The typical time to relapse in non-TNBC patients is 35–67 months, but that in TNBC patients is just 19–40 months. The death rate of TNBC patients within 3 months following recurrence is as high as 75% [23]. Therefore, the early-stage detection of TNBC is crucial for disease prognosis and treatment. Analysis of gene expression profiles often identify TNBC as a subtype of basal-like breast cancer, however, based on the recent molecular profiling, TNBC is divided into six categories: basal-like 1 (BL1), basal-like 2 (BL2), immunomodulatory (IM), mesenchymal-like (M), mesenchymal stem-like (MSL), and luminal androgen-receptor (LAR) expressing. Each subtype of TNBC has a distinct biology that responds differently to current therapy [24]. In this paper, except healthy breast sample, we used one non-TNBC subtype BT474 (luminal/HER2-positive), four types of TNBCs including HCC70 (BL2), HCC1806 (BL2), MDA-MB-468 (BL1), and MDA-MB-231 (MSL) for the study. The methodology, measurements, and biomarkers presented here can provide a new insight for the subtyping of breast cancers and aggressiveness grading.

The Kerr Effect is a nonlinear material response to an intense optical pulse. The pulse alters the material’s refractive index by populating various material states and distortions. These responses all have different magnitudes and response times that are dependent on the structure, properties and composition of the sample being optically pumped. No two materials have the same Kerr response. The subtle changes in dielectric and conductive processes produced when a tissue becomes diseased are shown hereto be enough to be detectable by a femtosecond Kerr Gate.

### THEORY:

The Optical Kerr Effect describes how an intense laser pulse causes the formation of a new temporary term in the expression of a material’s refractive index that is aligned along its electric field [1–4,25–28], such that,

1 $$n\;\left(t\right)=n_{0}+n_{2}I\;\left(t\right),$$

where *n* (*t*) is the total refractive index expressed by the material, *η*_0_ is the normal refractive index, *I* (*t*) is the laser intensity, *n*_2_ is the Kerr index, and *t* is time. The term n_2_ is made up of several contributing processes giving.


2 $$n_{2}=\sum_{i}n_{2}^{\left(i\right)}.$$

Each n_2_ process has its own response time $\left(\tau_{i}\right)$ and magnitude. To account for this the change $\left(\delta_{n}\left(t\right)\right)$ in the refractive index due to the Kerr Effect can be written as:

3 $$\delta n\;\left(t\right)=\sum_{i}\frac{n_{2}^{\left(i\right)}}{\tau_{\left(i\right)}}\int_{-\infty }^{t}I\left(t\right)\;e^{-\left(t-t^{\prime }\right)/\tau_{\left(i\right)}}dt^{\prime },$$

with $I\;\left(t\right)=I_{0}e^{-4\ln2\left(t^{2}/\tau_{L}^{2}\right)}$, *I*_0_ as the peak intensity, and $\tau_{L}$, is the laser pulse duration. In the transient condition, where $\tau_{L}<\tau_{\left(i\right)}$, the integral in Eq. 3 trend toward zero. This condition means that when using a sufficiently small pulse long index contributions become negligible. This is why femtosecond lasers are largely limited to: electron cloud distortions $\left(\tau_{\left(e\right)}\approx 10^{-17}\text{s}\right)$, molecular mechanisms $\left(\tau_{\left(m\right)}\approx 10^{-13-12}\text{s}\right)$ [1,4,25,27], and plasma generation $\left(\tau_{\left(p\right)}\approx 10^{-14}\text{s}\right)$ [29–31]. The exact values of these response times are unique to the state and composition of the material being optically activated.

A recent study done in human and animal tissues found that molecular mechanisms $n_{2}^{\left(m\right)}$ are small in tissues for femtosecond pulses, leaving only electronic and plasma mechanisms. The plasma index, $n_{2}^{\left(p\right)}$ is negative, meaning that it counteracts the other Kerr mechanisms. Expect at very high input intensity, the plasma index contribution is typically very small [27], however in femtosecond pumped tissues, where the molecular mechanisms are negligible, the plasma ionization index can be as strong as the weakly populated electronic index. The interplay between electronic and plasma indices gives tissues a unique δn(t) that will vary from positive to negative.

A Kerr Gate can measure δn(t) by using the fact that the index change will be aligned along the electric field of the pumping laser. This causes the material to become temporarily birefringent and capable of imparting a phase change (δφ(t)) on a secondary probing beam. Much like a waveplate or uniaxial crystal δφ(t) can be calculated using,

4 $$\delta \varphi \;\left(t\right)=\left(2\pi \frac{L}{\lambda_{1}}\right)\;\delta n\;\left(t\right),$$

where, *L* is the length of the interaction and λ_1_ is the wavelength of the probing beam.

A Kerr Gate measures phase changes, δφ(t), as a function of time delay between the pumping and probing pulse. The phase change, δφ(t), can be measured passing the probe beam through two crossed (π/2) linear polarizers with the sample in-between. In this configuration, no light from the probe can pass through the second polarizer except when a phase change is produced in the sample. Varying the path length of one of the beam arms can produce temporal delay; such the probe pulse can be made to arrive before, behind, or in unison with the pump. The measured path length change can then be converted into a time delay between the pulses. By measuring the signal from the second polarizer at different time delays a series of “snapshots” can be taken to produce a curve representative of the populations and lifetime of δφ(t). A Kerr Gate can also not discern between a positive of negative rotation, so the signage of δφ(t) is lost. This is why the δn(t) of tissues, which varies from positive to negative, appears as double peaks.

Mathematically the signal from the Kerr Gate can be calculated by convolving the probes pulse temporal profile with a gating function that produces a value between 0 and 1 as δφ(t) morphs between 0 and |π| [1,5,25,27].


5 $$S_{t}\;\left(\tau_{D}\right)=\frac{1}{S_{1}}\int_{-\infty }^{\infty }I_{Probe}\left(t-\tau_{D}\right)sin^{2}\;\left(\frac{\delta \varphi \left(t\right)}{2}\right)\;dt.$$

Here $S_{t}\;\left(\tau_{D}\right)$ is the time delayed signal from the second polarizer, *S*_1_ is a scaling factor and $\tau_{D}$ is the temporal delay relative to the pump pulse. One limitation of a Kerr Gate is that in the event that δφ(t)>|π| the signal will become oscillatory as the probe beam will be able to pass through the second polarizer multiple times over the course of the interaction [1,25,27]. When this method was previously applied to tissues using femtosecond pulses, the Kerr Gate produced a unique ultrafast dual peak structure, which suggested it was produced entirely from electronic and counteracting plasmas mechanisms.

The relaxation time of the plasma associated Kerr response is related to the dielectric relaxation time $\left(\tau_{r}\right)$, which describes the ability of free electric charges to move around in a material due to an applied electric field given by Eq. 6 [32]:

6 $$\tau_{r}=\frac{\epsilon_{0}n_{0}^{2}}{\sigma },$$

where, $\epsilon_{0}$ is the permeability of free space, *n*_0_ is the index of refraction, and σ the conductivity.

The dielectric and conductive properties of a biological tissue are based on its type, components, structure, living or non-living state, healthy or diseased, and physiology [33–35]. A change in these *n*_0_ and σ properties will produce a different relaxation time, which could be measured using an Optical Kerr Gate.

The observed Kerr plasma fall time $\left(\tau_{k}\right)$ and the dielectric response time $\left(\tau_{r}\right)$ are related to each other through Eq. 7:

7 $$S=e^{-t/\tau_{k}}=\delta n^{2}=e^{-2t/\tau_{r}},$$

where *S* is the Kerr signal intensity, and δn is the Kerr index change. From Eq. 7, the following can be deducted:

8 $$2\tau_{k}=\tau_{r}=\frac{n^{2}\epsilon_{0}}{\sigma },$$

Using the measured fall time of the Kerr plasma response in tissues (second peak in time) the conductivity of the tissue can be calculated using Eq. 9:

9 $$\sigma =\frac{n^{2}\epsilon_{0}}{2\tau_{k}}.$$

The conductivity change between healthy and cancerous tissues is shown here to be strong enough to produce measurable changes in the Kerr response indicating that conductivity is a new biomarker for cancer and its grade.

## METHOD

**Figure 1:**
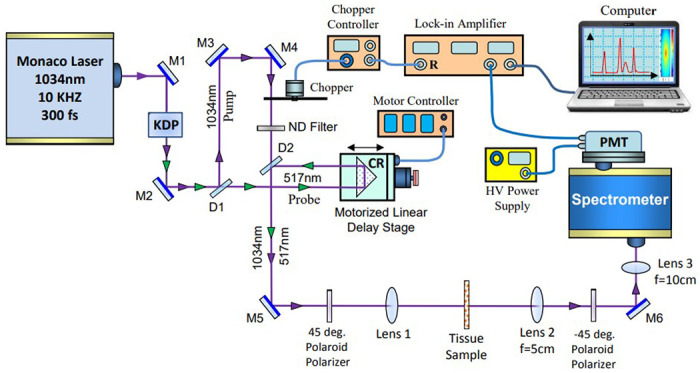
Schematic of the femtosecond optical Kerr Gate experimental set-up used for measurements to test various types of normal and breast cancer tissues. Key: M-mirror, ND-neutral density filter, CR- corner reflector, PMT-photomultiplier, D-Dichroic mirrors, and Lens 1 is a interchangeable lens. All data presented here was taken with Lens 1(f) = 2.5 cm.

The Kerr Gate was constructed using a Coherent Monaco Laser System. The 1034 nm 10 kHz 280 fs output of the system was frequency doubled to 517 nm 160 fs and the two wavelengths were frequency separated by a dichroic mirror into a 4.9uJ 1034 nm pump and 0.25 uJ 517 nm probe arm (Fig. 1). The pump arm passed through a 1696 Hz chopper and its intensity could be modulated by a neutral density filter. The probe arm is aligned through a corner reflector atop a motorized delay stage that can make 0.05 mm adjustments to the probe’s path length, which translates to time delay between the pump and probe pulses. Both arms recombine at a second dichroic mirror and travel collinearly into a polaroid plastic sheet linear polarizer. The polarized is invisible to the pump wavelength and imparts of polarization of 45o on the probe with respect to the pump. The beams then focus using a 2.5 cm lens into the sample location. Light leaving the sample is collected and collimated by a 5 cm lens and passed through a second linear polarizer at −45o, such that no light from the probe passes through except when a phase rotation is being produced in the sample, creating a Kerr Gate. Light that passes through the second polarizer is focused by a 10 cm lens into the slight of a single grating spectrometer set to 517 nm and photo multiplier tube (PMT). Electrical signals from the PMT are analyzed by a lock-in amplifier frequency tuned to the chopping frequency. This method of lock-in detection dramatically increases signal to noise level by only amplifying probe signals that are occurring at the chopper set pump frequency. Spatial and temporal overlap between the beam arms were originally found by Kerr Gating a 1 cm thick piece of glass, and later confirmed using a 1 cm cell filled with CS_2_.

The tissues studied consisted of various types of mouse breast cancer (non-TNBC and TNBC grades) and a pair of in vitro human breast tissues (cancer and normal). The healthy and cancerous human breast samples were obtained post-mortem and were supplied by the National Disease Research Interchange and the Cooperative Human Tissue Network under an Institutional Review Board (IRB) protocol. These samples were cut while frozen with an industrial standard razor blade; its 1mm thickness was measured with a digital caliper with an accuracy error value of ± 0.25 mm. After being cut they were kept in a phosphate-buffered saline (PBS) solution.

The mouse Non-TNBC and different subtypes of TNBC samples are obtained from Shi Lab in University of California, San Diego. The mouse samples were performed in accordance with protocols approved by the Institutional Animal Care and Use Committee (IACUC) of Memorial Sloan Kettering Cancer Center and the IACUC of the City College of New York and University of California San Diego. These fixed breast cancer samples were processed with coronal section by using a compresstome at about 1 mm thickness. They were stored in a phosphate-buffered saline (PBS) solution after being sliced. The 1 mm breast tissue samples were removed from the PBS to be placed natively (no glass) in a circular aperture mount of about 8 mm in diameter. Each sample was scanned a minimum of three times to test reproducibility of the signal. The laser operated at a fluence of approximately ~ 0.06 J/cm^2^, significantly below the expected damage threshold of biological tissues (2 J/cm^2^) [36]. Due to the chopper the repetition rate of the pump is low enough to neglect the possibility of thermal damage. The experiment performed and the handlings of the post-mortem human tissues were approved by the IRB following the guidelines and regulations of the Human Research Protection Program at City College CUNY.

## RESULTS

**Figure 2:**
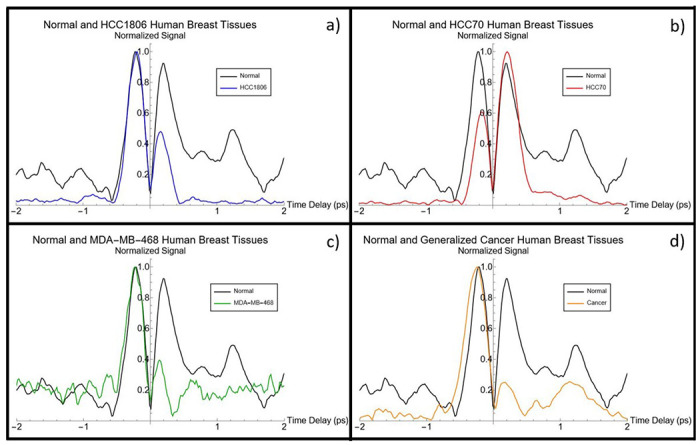
Kerr Gate signal comparison for normal and cancerous tissues. Kerr Gates of normal (black) compared to a) TNBC sample HCC70 (red), b) TNBC sample HCC1806 (blue), c) TNBC sample MDA-MB-468 (green), and d) Generalized Cancer (orange). Taken at input pump pulse energy of 4.9 uJ. Exponential rise and fall times can be found in Table 1. Sample thicknesses were about 1 mm free standing.

The resulting temporal Kerr Gate signals for normal, generalized cancer, and different TNBC tissues can be seen in Fig. 2. Additional gates were performed other TNBC samples not shown in Fig. 2. All gates displayed the unique dual peak structure that had been previously reported in avian breast and human brain tissues [7]. To understand how these Kerr gate signal corresponds to changes in the Kerr index both of the peaks seen in the resulting signals can be fitted to exponentials to get their rise and fall times, which can be found tabularized in Table 1 for all tested samples.

As can be seen in Table 1 some fairly dramatic changes occur in the rise and fall times of the Kerr Gates between samples. Most TNBC samples have a shorter peak 1 rise time than that of normal tissue, while Cancer, MDA-MD-468 (TNBC), and BT474 (non-TNBC) all have longer rise times. The fall times of peak 2 also sees the most significant change. Normal healthy breast tissue has a much longer fall time than all of the cancerous and TNBC samples, with that difference ranging between + 132–196 fs. Cancerous tissue and non-TNBC BT474 have fall times around 162 fs and all TNBS samples fall to within 100–142 fs. Interestingly, the rise and fall times for the sample of generalized cancer are nearly identical to that of non-TNBC BT474, which suggests that the two samples may be related.

Signal to noise ratio would vary between scans and samples. Samples with the highest ratios were HCC70 and HCC1806 (Fig. 2) and the sample with lowest ratio was generalized cancerous tissue (Fig. 2d). There may be several factors for this, for example, freshness of sample, water content, age, and physical health of donor. These could impact aspects such as absorption and scattering during the gating process. The ratio of the peak strength also changes between samples when performed at the same pump pulse energy, however it is difficult to draw a conclusion form this as the ratio would occasionally change between successive scans, likely due to the sample drying out or fluctuating in temperature. Rise and fall times of the peak stayed roughly constant for each sample between successive scans. “Switching” position, where peak 1 transition to peak 2, see some movement between samples but is likely due to minor changes in temporal overlap that could occur due to laser drift. Healthy breast tissue exhibited a 3rd peak occurring at approximately 1.3 ps. This peak was consistent between all four scans of the health tissue, and its late timing suggests that may be a weak representation of slower molecular effects that could be attributable to structural differences between the normal and cancerous state. This statement requires more study.

## DISCUSSION

The temporal structure of the Kerr Gate agrees with previously reported data in human brain and avian breast tissue [7], suggesting that future work done on both human and animal tissues using femtosecond pulses can expect to find comparably structured gates. This double peaked structure, based on both its speed and shape is arising from interplay between the electronic (peak1) and negative plasma response (Peak 2). Likewise, the maximum phase change produced should fall to within δφ(t)≤|0.13π|. Longer pump pulses may allow for the activation of slower molecular mechanisms fundamentally changing the gate temporal structure and strength.

The rise and fall times of both peaks translate to the rate of change in the Kerr index as it transitions between its ultrafast electronic mechanism (peak 1) and its slower counteracting plasma mechanism (peak2). These values see significant change between samples. The rise time of peak 1 and the fall time of peak 2 see the largest fluctuations and suggest that they could potentially be used in optical biopsy. The rise time of peak 1 is shown to have some variance between samples. Cancer BT474, and TNBC MDA-MD-468 all have longer rise times than that of normal tissue, while all other TNBC’s had shorter. The variance here suggests the electronic cloud interacts with the pump laser pulse at different rates depending on the sample’s composition.

The primary change comes in the form of the response time of the second peak with its representative of the negative contribution of plasma and ionization. As seen in Table 1 the fall time of peak 2 for normal tissue is significantly longer than all other tested samples, including cancer, by 130 to 196 fs (an 81 to 194% increase). These values all fall to within the expected range of plasma relaxation [29–31]. Following Eq. 9, the variance observed in peak 2’s fall time, suggests a fundamental change in the tissue’s dielectric and conductivity properties when infected with cancer. The dielectric constant and conductivity are already known to differ between organs and types of brain matter [33], so a similar change would be unsurprising for diseased tissues.

The information given in Table 1 from peak 2 for the fall time along with Eq. 9 can be used to create approximated calculations of the different tissues’ conductivity as shown in Table 2. To calculate the conductivity (Eq. 9) for the different samples, the index of refraction of normal breast tissue was estimated to be *n_normal_* = 1.345, and cancerous was estimated at *n_cancer_* = 1.359[37].

As shown in Table 2, the dielectric response time $\left(\tau_{r}\right)$ is twice Kerr fall time $\left(\tau_{k}\right)$ observed in peak 2. After finding $\tau_{r}$, the conductivity (σ) in tissue for healthy and cancer breast tissue can be calculated for each type, where the conductivity with the highest values (64.9, 57.6, 70.5 81.8 S/m) are obtained for the more aggressive type of breast cancer (TNBC). This change in *σ* is related to the different relaxation times as deducted from Eq. 8 and Eq. 9. The significant change in *σ* can be attributed to the healthiness of the breast tissue and the abnormality of the cancer cells. The overall results indicate that the conductivity found in the tissue is a key fingerprint for breast cancer classification.

The third peak present in healthy tissue Kerr Gates, which can be seen as black in Fig. 2, marks an additional change between healthy and cancerous breast tissue, potentially due to the presence of slower molecular n_2_ contributions. The difference may be associated with structural changes in the tissue’s extracellular meshwork matrix based on the compositions of collagen and elastin found in the breast. The timing of the signal suggested that healthy tissue is able to weakly exhibit the longer molecular contributions to the Kerr effect in response to a 280 fs pulse, which could not be seen in any of the cancerous tissues. The signal to noise ratio of peak 3 makes it difficult to conclusively assess its origin, but its presence could be seen in all four tests of healthy tissue. This observation would suggest that healthy tissues have different molecular pathways, as they relate to the tissue’s matrix structure, giving it a stronger and faster molecular response to an ultrashort laser pulse. This would enable it to survive the integral relationship between pulse duration and response time described by Eq. 3, allowing for its limited presence in Fig. 2 [4,17].

## CONCLUSIONS

Kerr Gate temporal signals are used for the first time to measure the nonlinear Kerr index response of healthy and various grades of cancer for the purpose of optical biopsy. Temporal differences in the sample types display meaningful changes in the rise and fall times of the multiple peaks associated with the Kerr response. Observations of the rise time of the first peak suggest minor changes in the rate in which different tissues’ electronic clouds deform in response to an electric field. The largest changes are observed in the decay of the second peak, which changes substantially between healthy and infected tissues while also varying with cancer grading. Since the second peak corresponds to the plasma (ionization) response of the tissue, this suggests that there is a significant change in the dielectric and conductivity properties of the tissue once infected with cancer. Using the fall time of the second peak the conductivity could be calculated, suggesting the cancerous tissue is 2 to 3 times more conductive than healthy breast. Data shown in Fig. 2 also suggests that slower molecular contributions are also weakly active in healthy tissue, but not in cancerous, in response to a femtosecond pulse. This can all be explained by cancer related distortions in the tissue’s extracellular matrix, which increase charge flow but restrict molecular motions. The Kerr Gate method described here could potentially be used as a new application in optical biopsy and cancer science studies. In general, the dielectric property of the tissue such as the conductivity can be proposed as a tool for characterizing or classifying diseases linked to tissue type and structure.

The significance of this result warrants replication of this method in other tissues and diseases. Other cancers (bone and brain) should be investigated to see if they produce the same change in the temporal plasma response, as well as other diseases such as Alzheimer’s. Since this method is reliant on transmission through the tissue, a backscattering Kerr Gate should be attempted to produce a potentially less invasive method that could be used on a live patient. The effect of pulse duration should also be explored. Longer pulse durations such as those of picosecond lasers may exhibit the molecular Kerr response, which could display additional diagnostic properties. As shown here the conductivity of the tissue is a key diagnostic fingerprint for cancer and disease detection using OKG.

This work points to other standard methods to measure the electric conductivity in tissue such as from direct current (DC) and from radio frequencies (RF), and optical frequency regions (GHz, THz) using the principles of time-domain reflectometer [38]. Through these methods one can measure the disease state of tissue such as cancer, which its early detection improves survival rates [39]. In general, the dielectric properties of tissues can be used as a diagnostic method to find illnesses present in a tissue [35].

## Figures and Tables

**Table 1:** Exponential rise and fall for normal and cancerous samples. Exponential rise and fall times of the dual peak Kerr Gate structure produced in all tested tissue samples.

	Peak 1		Peak 2	
Sample	Rise (fs)	Fall (fs)	Rise (fs)	Fall (fs)
Normal	126	90	110	294
Cancer	152	120	64	162
HCC70	84	78	100	126
HCC1806	94	90	62	142
MDA-MB-231	100	64	100	116
MDA-MB-468	168	64	104	100
Non-TNBC BT474	198	116	72	162

**Table 2:** Dielectric response and conductivity for normal and cancerous samples. Kerr relaxation time and the tissues conductivity based on the dielectric response time.

Sample	Peak 2 Fall $\left(\tau_{k}\right)$	Dielectric response time$\left(\tau_{r}\right)$	Conductivity (σ)
Breast Tissue	(*fs*)	(*fs*)	(S/m) or (Ωm)^−1^
Normal	294	588	27.2
Cancer	162	324	50.5
HCC70	126	252	64.9
HCC1806	142	284	57.6
MDA-MB-231	116	232	70.5
MDA-MB-468	100	200	81.8
Non-TNBC BT474	162	324	50.5

## Data Availability

Any data will be available upon reasonable request made to corresponding author Henry J. Meyer hmeyer000@citymail.cuny.edu.
